# Entropy Crises in Glasses and Random Heteropolymers

**DOI:** 10.6028/jres.102.014

**Published:** 1997

**Authors:** Peter G. Wolynes

**Affiliations:** School of Chemical Sciences, University of Illinois, Urbana-Champaign, Urbana, Illinois 61801 USA

**Keywords:** entropy crises, glass transitions, random first order transitions, random heteropolymers

## Abstract

The concept of random first order transitions with configurational entropy crises provides a theoretical framework for understanding the glass transition. This paper discusses such transitions in exactly solvable spin glass models and in globular random heteropolymers and their relation to glass transitions in molecular fluids and polymers. The Vogel-Fulcher law is shown to be related to the search time through the energy landscape of an “entropic droplet.”

## 1. Introduction

Watch the Entropy! Albert Einstein is said to have remarked that he was able to imagine the overthrow of all of the laws of physics except for the Second Law of Thermodynamics. Such a statement seems surprising to most people for whom the Second Law and its central mathematical quantity, the entropy, are among the most abstract concepts. Perhaps entropy’s primacy in Einstein’s mind was made more vivid through the discovery of the Third Law of Thermodynamics by Einstein’s experimental colleague, Nernst. The Third Law which states that the entropy of any system may be taken to be zero at the absolute zero of temperature was a foreshadowing of quantum statistical mechanics. The quantum theory allows us to realize that entropy is no more complicated than counting. The interpretation of entropy as the logarithmic count of quantum states makes the Second Law transparent and if, unlike the older Einstein, we embrace wholeheartedly the quantum idea, it also makes the Third Law of Thermodynamics one we can count on too. This is why watching the entropy can be such an important clue to the behavior of systems. Many times in low temperature physics, failure to account for all of the entropy at higher temperatures signalled emergence of new phenomena and even new phases as the temperature was lowered further towards absolute zero.

The first entropy crisis occurred in 1931. By measuring the heat capacities of supercooled liquids, Simon showed that simple extrapolation of the heat capacity to absolute zero would give a negative entropy violating the third law of thermodynamics [1]. In fact, the Third Law was always saved by the intervention of the glass transition so that the low temperature amorphous phase had a residual positive zero point entropy as interpreted by Pauling [2]. Residual entropy became a classic problem for undergraduate physical chemistry students. It was Kauzmann in 1948 who pointed out a possible paradox in these results [3]. Supposedly the glass transition was a purely kinetic phenomenon. If so, it is simply a matter of impatience which prevents the experimentalist from measuring the equilibrated heat capacity at low temperature for the supercooled liquid. Something had to happen in order to prevent the crisis in the third law, the entropy crisis, in this case. Kauzmann’s study, as well as many later ones notably by Angell and coworkers [4], make clear that a very rapid variation in the entropy must occur at some temperature below the kinetic glass transition in order to avoid this entropy crisis. It is natural to expect then that the slowing down which occurs at the glass transition as manifested in the non-Arrhenius temperature dependence of kinetic properties is somehow tied to this “ordering” which may, in fact, be a phase transition.

Polymers have provided some of the most important examples of glass transition phenomena and motivated much theory. In 1958, Gibbs and diMarzio developed an approximate theory of dense melts of homopolymers with an energetic bias for extended configurations of the chain [5]. Their approximate treatment of the statistical mechanics of this model led to an entropy crisis and seemingly a phase transition at a finite temperature above absolute zero. Their theory has been extended in many ways to more complex polymer systems and accounts for many of the qualitative trends of the glass transition with chemical structure and the effects of plasticizer. Later Adam and Gibbs produced an argument for the super Arrhenius temperature dependence of transport properties based on the entropy crisis in this model [6]. The gist of their argument is that even for the low entropy density state, a region of the glass or viscous liquid must have more than one state in order to reorganize; therefore, the volume of a rearranging region must be inversely proportional to the entropy density. By making the hypothesis that the activation energy for this event is proportional again to this volume, they obtained a super Arrhenius temperature dependence that would lead to complete dynamical arrest at the ideal glass transition temperature. Furthermore the explicit dependence of the apparent activation free energy on the entropy density has been confirmed in many later studies of polymers. Over the years the ideas of the Kauzmann paradox, the possibility of an ideal thermodynamic glass transition and the connection of these to kinetics have remained controversial notions, to say the least, among theoretical chemists and physicists. There are several reasons for this: Foremost is the direct unobservability of the phase transition to the ideal amorphous state by supercooling the liquid. (The dynamical arrest seems like a “Catch 22” argument to some.) Nevertheless, over the years, the empirical case has become, I believe, much more convincing because of the preponderance of the experimental evidence. The theoretical arguments have not convinced people entirely either; several objections have been raised: The Gibbs/diMarzio treatment of the polymer entropy is not exact but approximate. Other approximate treatments of the same model suggests the absence of an entropy crisis for the homopolymer system. The Adam/Gibbs kinetic argument has its own stylistic problems, despite its elements of naturalness. The energetics of activation in the argument do not come from the same Hamiltonian that gives the underlying transition. They seem to be grafted on to the analysis. To some extent this is a natural feature for any kinetic theory, but there are certainly other problems such as nucleation of crystals from a melt where few additional energetic assumptions need to be made. Testing the argument directly has been difficult since the size of the clusters from the Adams/Gibbs argument is so small that they would again be essentially unobservable, even in the best case. Again, this has detracted from the acceptance of the basic idea of tying a kinetic phenomenon to an underlying equilibrium transition.

In this paper, I review the theoretical work of the last decade which in my view removes most of the conceptual objections to an entropy crisis as the underlying basis of the glass transition [7]. The crucial observation is the existence and, indeed, widely universal behavior of a class of systems that possess entropy crises of the type envisioned for structural glasses, but for which the statistical mechanical problem can be solved exactly. These models can still be objected to as containing quenched randomness and extremely long-range interactions. While these remain objections to the use of these models for explaining the glass transition of simple molecular fluids, they are less powerful objections when applied to polymeric glasses. Polymeric glasses are most easily formed from atactic polymers in which random stereochemistry applies at each assymmetric center. The atacticity implies, at some level, that the glassy polymers are actually heteropolymers with a quenched sequence. The interactions are random due to the diastereomeric interaction of the subunits. In addition, some long-range interactions are present because of the high molecular weight of the polymer and its chain connectivity. I, myself, do not believe the randomness and long range interactions of the exactly solved models are essential for the universality of the glass transition phenomena but I believe for polymers, at least, the presence of them is a sound counter argument to the usual objections. The dynamics of these random models is still quite complex [7,8,9,10]. In the meanfield limit, they can also be shown mathematically to be connected with the mode-coupling theories which have been used to study atomic glasses [11] and polymers [12]. Also for finite systems, kinetics for activated transitions can be written down and the kinetic models solved explicitly for some of the simpler cases [13]. None of these more or less explicit treatments of dynamics lead to the Vogel-Fulcher law behavior, however.

Nevertheless, plausible arguments very much analogous to those used for metastable systems with first order transitions provide a route to the Vogel-Fulcher law in models with finite range interactions [9,14]. These arguments which explain directly how the activated state is related to the ground state provide an alternative to the Adam/Gibbs approach which separately treats the activation energetics from the ground state thermodynamics. The droplet size predicted from these arguments is larger and therefore are more consistent with recent experiments on finite size effects in glasses and on spatial heterogeneity of highly viscous liquids.

The discussion is organized as follows: In the next section I introduce the class of random systems which exhibit random first-order phase transitions. Following this, I discuss two members of this class. One of these is the famous random energy model of Derrida, whose analysis is extremely straightforward and gives the simplest treatment of the underlying transition. I will also discuss the generalized random energy model and its application to a heteropolymeric globule. This simple model shows the emergence of a dynamical transition connected with the thermodynamic one. I explain the set of arguments needed to treat systems with finite range interactions and show the connection of the thermodynamic transition with the Vogel-Fulcher law. Finally I summarize and describe how these ideas can be important conceptually for understanding the kinetics of protein folding.

## 2. Random Models With Entropy Crises

Entropy crises struck random models very early in their development. Magnetic alloys with spins on dissolved impurities exhibit some phenomena vaguely reminiscent of glasses. When cooled, their dynamics become sluggish and the spin orientations remain trapped for very long periods of time. Such magnetic alloys were called spin glasses by Edwards and Anderson [15]. The varying distance between the dissolved spins leads to interactions which are, at random, both ferromagnetic and antiferromagnetic. It is for this reason that no simple-to-describe ordered state is formed at low temperature. The interactions are said to be frustrated since both the ferromagnetic and antiferromagnetic tendencies cannot simultaneously be satisfied. While the spins, therefore, form an amorphous frozen state, the detailed features of the phenomenology of these spin glasses differ dramatically from the ordinary glasses. For instance, they do not exhibit a change of heat capacity upon freezing as liquids do and experimentally, they do not exhibit the signs of an impending entropy crisis. The meanfield theory of spin glasses did, however, bring out an entropy crisis at first. A meanfield spin glass is one in which the spins are imagined to interact with random terms but of very weak and very long range. The Hamiltonian for such a system can be written as,

$$H=-\sum J_{ij}S_{i}S_{j}$$ (1)

where the *j_ij_* are Gaussian random variables. This is the so-called Sherrington-Kirkpatrick model spin glass [16]. Historically the randomness of the spin glass Hamiltonian made the meanfield theory for it difficult. An elegant formal technique was introduced for averaging the free energy over the random interactions [17]. This technique involves some mathematically obscure steps but the method has no serious ambiguity when correctly applied. The ultimate results of the technique, called the replica method, have been checked against computer simulations and have been shown to be correct. The replica technique consists then of formally evaluating the average of the logarithm of the partition function which gives the free energy by considering n copies or replicas of the system with the same random interactions.

$$\langle \log Z\rangle =\underset{n\to 0}{\lim}\langle \frac{Z^{n}-1}{n}\rangle .$$ (2)

The average over the random interactions leads to an effective coupling between the different copies which were treated by standard effective mean fields à la Weiss’s theory of ferromagnetism. This coupling may be somewhat counter intuitive since we said the replicas were simply independent copies of the same system, but we must remember they have the same interactions. Thus there will be a tendency for each of the systems to go into similar configurations. This would be interpreted after the average is taken as an effective interaction between the copies. A useful analogy concerns children playing on a street in the summer. When the ice cream truck comes, they all go towards it and are brought together as if they were attracted to each other but, in fact, they are all independently seeking the same satisfying situation. This effective interaction is what gives rise to the phase transition. The only thing that is peculiar in the mathematics is that at the end of the calculation, one takes the limit as the number of copies goes to zero! This is what leads to the mathematical peculiarities. When Sherrington and Kirkpatrick first tried to solve the problem by what were the standard meanfield methods, they encountered an entropy crisis. Their solution, which was sensible near the transition, gave a negative entropy at absolute zero. Later, following a clue of deAlmeida and Thouless, Bray and Moore improved upon this meanfield approximation by using the notion of a broken replica symmetry [18]. This was the idea that the replicas need not be all identical, but that they could be broken up into groups, some of which were highly similar but some of which had lower degrees of similarity. The simplest scheme of doing this improved the entropy crisis but did not cause it to disappear entirely. It was only later when Parisi used the replica symmetry breaking trick in a hierarchical fashion that a meanfield solution satisfying the third law of thermodynamics was obtained [19]. It is this solution which stands the test of comparison with computer simulation.

The transition predicted by this mean field approach is continuous. The different copies of the system have only a very mild similarity when the transition begins but this similarity continues to grow as temperature is reduced, just like an ordinary magnet. It is this continuity which leads to the lack of significant heat capacity anomaly at the transition. There are other “spin glass models,” however [20]. These do not model the magnetic alloys well, but are mathematically analogous. We could, for instance, imagine a system in which the spins are not objects simply pointing up or down, but where they point in a larger but still discrete number of directions, say the corners of a tetrahedron. These are so-called Potts spins and the spin glass model can be generalized mathematically to treat these. Similarly one can imagine energy functions in which the spins still point up or down but in which the spins interact in triplets or even higher numbers of spins cooperating at a time.

$$H=-\sum_{p-\text{tuplets}}J_{ijk\ldots p}\;S_{i}S_{j}S_{k}\ldots S_{p}.$$ (3)

The technology of replica meanfield theory was applied to these more general spin glass energy functions which lack up/down symmetry [20]. These less symmetric models also show phase transitions but are first order in the sense that the similarity between different copies takes a jump at the phase transition. This leads to a discontinuity of the heat capacity and the magnetic susceptibilities at the phase transition. The similarity of these mean field asymmetric spin glass phase transitions to the liquid structural glass transition was noted first by Kirkpatrick and Wolynes [8]. Both the density functional theory of glass transitions [21] and mode-coupling theory of atomic liquid glass transitions, led to phase transitions that were not second order but had a discontinuity in the appropriate similarity order parameter like these random models. For the density functional theories, the order parameter was the mean square displacement of a molecule from its a-periodic lattice position. In mode coupling theory, the analogous quantity was the Lamb-Mossbauer factor which could be measured by neutron scattering. They also noted that the discontinuity at the thermodynamic transition of the Potts glass of the heat capacity resembled the extrapolation of the laboratory liquid glass transition down to a putative ideal thermodynamic glass transition temperature. The analogy was deepened when Kirkpatrick and Thirumalai showed that the mode-coupling theory could be applied to the *p* = 3 spin model [22] and gave exactly the mode-coupling equations that were used in treating structural glasses by Leutheusser.

This mode-coupling treatment, however, led to a puzzle. The mode-coupling treatment did not predict the dynamical transition at the same point as the thermodynamic transition. There seemed to be two different transitions in the model. An entropy crisis provided the key to the interpretation. None of the meanfield solutions for the Potts glass or the *p* = 3 spin glass exhibited directly an entropy crisis. The entropies were always positive and obeyed the Third Law. However, it was clear that the dynamical transition could be interpreted as an instability point. It was the point at which individual free-energy minima became unstable to thermal vibrations. It then became possible to count these minima in the temperature range below the dynamical transition and above the thermodynamic one. While this calculation is elaborate, the configurational entropy of these basins could be calculated and Kirkpatrick and Wolynes [9] showed that this configurational entropy continued to decrease until it reached zero at the replica calculation’s thermodynamic transition.

Thus we see a wide class of models with quenched randomness exhibit transitions that resemble the structural glass transition which has no *explicit* randomness in the Hamiltonian. The thermodynamic transition for all these models is related to a configurational entropy crisis.

The mathematical analysis of the general Potts spin glass or *p*-spin glass is quite involved. Much insight can be found by studying a simpler model called the random energy model [23,24]. This model assumes that individual configurations have random independently chosen energies and can be thought of as the limit of the *p*-spin model as *p* goes to infinity. A generalization of this model which allows the energy levels to be pairwise correlated, is also solvable. This is known as the generalized random energy model [25]. This system exhibits both the static and dynamic transitions like the more general case. The random energy model only exhibits the thermodynamic transition. We will discuss the thermodynamics and dynamics of these models explicitly in the next section.

We should also point out that the random heteropolymer falls in the same universality class as these models with random first-order transitions. The random energy model was used by Bryngelson and Wolynes [26,27] to describe the misfolded states of a protein, which should resemble a random heteropolymer. This led them to investigate the dynamics of the random energy model later. The formal techniques of replica meanfield theory were also applied to the random heteropolymer by Garel and Orland [28] and Shakhnovich and Gutin [29] confirming explicitly that random heteropolymers have random first-order phase transitions of the REM type.

## 3. Thermodynamics and Kinetics of the Random Energy Model and Its Correlated Generalization

The basic analysis of the random energy model is straightforward. Proving that the result is indeed exact and correct is not so simple. The rigorous proof by Derrida, who introduced the model, uses elaborate methods. Here we sketch the main idea. Each configuration of the system is assigned a random energy. While the model was first applied to a spin, the same approximation can be used for a large heteropolymer on a lattice. These systems have a finite total number of configurations, *Ω*_0_. The energy of each configuration is chosen at random. Like the other spin glass models, we imagine this energy comes from many conflicting terms added together. The central limit theorem then argues that the distribution function for this energy is a Gaussian,

$$P\left(E\right)=\frac{1}{\sqrt{2\pi \Delta E^{2}}}e^{-E^{2}/2\Delta E^{2}}.$$ (4)

It is tricky to calculate the thermodynamics of this model within the canonical ensemble, but with the microcanonical ensemble, it is straightforward. The density of states for a given energy is on the average,

$$\langle n\left(E\right)\rangle =\Omega_{0}P\left(E\right).$$ (5)

Notice that the density of states, when it is larger than 1, cannot fluctuate much so when ⟨*n*(*E*)⟩ is larger than 1, this is a good approximation to *n*(*E*)` for each specific choice for the random energies. The entropy, as a function of energy, can then be computed *S*(*E*) = *k*_B_log *n*(*E*), giving:

$$S\left(E\right)=k_{B}\log\;\Omega_{0}-\frac{E^{2}}{2\Delta E^{2}}.$$ (6)

To obtain the thermal properties, we must relate the temperature to the energy. This is done through the standard thermodynamic relationship that comes from the Second Law, 1/*T* = ∂*S*/∂*E*. In this case it gives us the relationship between the average energy and the temperature,

$$\bar{E}=-\frac{\Delta E^{2}}{2k_{B}T}.$$ (7)

The entropy at any given temperature is therefore given by

$$S=k_{B}\;\ln\Omega_{0}-\frac{\Delta E^{2}}{2k_{B}T^{2}}.$$ (8)

Notice that this entropy is plummeting to zero and reaches that value at a finite temperature, *T*_0_, above absolute zero. This is given by

$$T_{0}=\frac{\Delta E/k_{B}}{\sqrt{2\ln\Omega_{0}}}.$$ (9)

It would seem that the entropy would even go negative, but this is not so. Below this temperature, *n*(*E*) is of the order of 1 on the average and is no longer approximated well by ⟨*n*(*E*)⟩. Therefore, the fluctuations from the average behavior are apparent in any given system. At this point, the system will be frozen in a small number of low energy states whose number is sufficiently small so their entropy is insignificant. Thus below this point, the entropy vanishes and remains equal to zero. Thus we see that the heat capacity vanishes below the phase transition. The heat capacity shows a parabolic form above the transition and exhibits a discontinuity at the ideal thermodynamic glass transition. The system is trapped in a specific state so the similarity between different copies of the system analogous to the Lamb-Mossbauer factor discontinuously changes from zero to a finite value. In that sense the transition is first order. But the transition has no latent heat. We call such a transition a “random first order transition” because of the discontinuity in the similarity order parameter. It is thermodynamically second order according to the Ehrenfest classification. The behavior of the heat capacity is very much like that seen in structural glasses for the excess configurational entropy, which is well fit by the hyperbolic form. This leads to an entropy which vanishes linearly at the transition. Other susceptibilities will also show discontinuities at the transition.

Because the random energy model is so explicit about its topography, it is easy to count local minima and to describe the statistics of barrier heights [6]. A crude estimate to the barrier height is easy to obtain since the landscape is so rough. Most minima are surrounded by typical states with energy approximately zero. At a temperature *T*, however, the thermally occupied minima have energy \O(E,^−^) = − Δ*E*^2^/2*k*_B_*T* which therefore gives the typical activation barrier. Thus the typical escape time from a minimum is

$$\tau =\tau_{0}e\Delta E^{2}/{\left(2k_{B}T\right)}^{2}.$$ (10)

This behavior is stronger than the Arrhenius law and was used by Ferry to describe the viscosity of liquids and polymers long ago [30]. Ferry later used the Vogel-Fulcher law as providing a better fit to the data [31]. An important picturesque result comes when we notice that the quadratic law can only hold down to *T*_K_. At that temperature the activation barrier would saturate according to Eq. (1).

The relaxation time at *T*_K_ is

$$\tau =\tau_{0}e^{S_{0}/k_{B}}.$$ (11)

This is the same time as to search all the states. For a single heteropolymer globule we call this the Levinthal time. Levinthal argued that if there were no special kinetic paths a protein (which is after all a heteropolymer) would have to search all of its states to find the ground state [32]. This clearly takes too long—giving rise to the puzzle of protein folding. Much of the theory of protein folding is devoted to finding “loopholes” in the Levinthal argument.

The random energy model may seem too extreme. Configurations of a finite system with some structural similarity should have similar energies. Each state of more general system with a random first order transition is not a thermodynamic basin as in the REM but many individual sates form such a basin. This is the analog of the vibrational entropy of a glass. A more complicated model that takes into account the correlation in the energy landscape can be defined and solved. Such a model that takes into account pair correlations was introduced by Derrida and Gardner [25]. At its simplest, the model is defined through a few characteristics. First a similarity measure which finally becomes the order parameter, *q*, quantifies the structural similarities of two configurations. The energies of two states with similarity *q* are correlated Gaussian variables with a covariance ⟨δ*E*δ*E*_2_⟩ = Δ*E*^2^(*q*). This is specified by the underlying interactions. The last defining quantity is the entropy of states *S*(*q*), with a given similarity *q* to a specifically chosen one. Derrida and Gardner show how a Hamiltonian model can be chosen to fit these characteristics and then can be solved to give the thermodynamics. This technique can be applied directly to a finite size heteropolymer globule [33]. The energy of a heteropolymer globule depends on pairs of contacts. Each contact in heteropolymer would contribute equally to the variance of the energies. In the analysis of Plotkin et al. [33], the appropriate similarity measure between two globule configurations is *q*, the fraction of contacts which involve the same sites in each configuration. The pair correlation ⟨δ*E*_1_δ*E*_2_) is then Δ*E*^2^*q*. The calculation of the other defining quantity *S*(*q*) then resembles Flory’s theory of polymer vulcanization, since each contact in common decreases the entropy much as chemical crosslinks do in rubber. The thermodynamic behavior of the GREM that has the correlations of a random heteropolymer globule is slightly modified from those for the REM with the same total entropy and energy variance. There is a random first order phase transition at a temperature only a few percent different from the REM estimate. The size of the basins [33] is substantial; then vibrational entropy is 3/4*S*_o_, however. It is the configurational entropy counting these basins which vanishes at the transition.

The kinetics of the GREM is more interesting because of the finite size of the basins [34]. The similarity measure *q* acts as a reaction coordinate for escape from any local minimum. The free energy profile for escape from a basin centered around a state of energy, *E_i_*, has the form

$$F\left(q;E_{i}\right)=-T\left[S_{0}\left(q\right)-\frac{\Delta E^{2}\left(1-q\right)}{2T^{2}}\right]-\frac{\Delta E^{2}}{T^{2}}\left(1-q\right)+qE_{i}.$$ (12)

The barrier height can be computed for each *E_i_* and the escape time averaged as in the theory of the REM dynamics. When the entropy is a piecewise linear function of *q* (as fits large heteropolymer globules) the analysis is relatively simple. At temperatures above a certain temperature, *T*_A_, the free energy profile for the average state is downhill. Thus escape from a basin requires no activation barrier. Below *T*_A_, however, dynamics becomes activated for a large fraction of the states and one obtains a modification of the Ferry law.

At the thermodynamic transition temperature the escape rate is considerably diminished from the Levinthal estimate. It scales in an exponential way with the configurational entropy, but with a smaller prefactor in the exponent. This again approximates the search time through the large basins, rather than the complete set of individual states. When applying this analysis to a protein this argument suggests that correlations do provide a significant quantitative amelioration of the search.

## 4. Entropic Droplets in Random First Order Transitions

Both the random energy model and its (globally) correlated generalization, the GREM have extremely long-range interactions so the entire system must reconfigure to escape from a basin. Thus the barriers for reconfiguration scale with the size of the system *N* at temperatures between *T*_A_ and *T*_K_ giving divergent time scales in the thermodynamic limit of a bulk material. The analogous treatment of the kinetics of an ordinary first order phase transition also gives barriers for converting from one phase to another that scale with system size. This is correct for a sufficiently small cluster of material undergoing a phase change but not for a bulk material where only a small region needs to change phase, following which it is easier for the rest of the system to change over, in that case by growth of the critical droplet. The nucleation barrier for such a metastable state ordering does not scale with system size but is finite. Local rearrangements at a random first order phase transition also provide a mechanism of escaping a local minimum with a finite size barrier.

What determines the size of the region which rearranges near a random first-order transition? The configurational entropy is extensive above *T*_K_. The entropy density is *s*_o_, therefore a region of size *ξ* in any global minimum state would enjoy a free energy advantage of magnitude − *k*_B_*Ts*_o_*ξ* to rearrange. On the other hand at the border of such a region, there would be a mismatch, giving an energy cost *Jξ^y^*. Naively we would expect this to depend on the surface area so we expect *y* = *d* − 1 = 2 in three dimensions. Since *d* > *y*, it is favorable for the system to form a mosaic with a correlation length *ξ* = (*J*/*k*_B_*TS*_c_)^1/(^*^d^*^–^*^y^*^)^. Each of the regions of the mosaic is in a low free energy configuration and is unstable at its edges where it meets a different region, itself in a low local free energy state. The barrier to rearrange is of the order *k*_B_*Ts*_c_*ξ^d^* ≈ *s*_c_^−^*^y^*^/(^*^d^*^–y)^.

The naive estimate for the mismatch energy in the mosaic thus gives a barrier scaling like (*T* − *T*_K_)^−2^ in three dimensions. This result was obtained by Kirkpatrick and Wolynes [9] initially and more recently by Parisi (35).

The naive mismatch energy is not consistent with the idea of there being only a single length scale characterizing the mosaic [7]. The discontinuity of the heat capacity suggests by the usual scaling arguments at a thermodynamically second order transition a correlation length scaling like (*T* − *T*_K_)^−2/^*^d^*. For consistency, this implies *y* = *d*/2. This mismatch energy scales exactly like the randomness energy of a region. According to scaling, each region of the mosaic looks like a random energy model precisely at its entropy crisis. Therefore the rearrangement time scale is

$$\tau =\tau_{o}e^{s_{o}}≃\tau_{o}e^{s_{o}\xi^{d}}≃\tau_{o}e_{s_{o}}^{\underline{A}}$$ (13)

The Vogel-Fulcher or Adams-Gibbs behavior is obtained. The argument here is distinct from that of Adams and Gibbs since there is a very different correlation length in the Adams-Gibbs theory *ξ*_AG_ ≈ (*T* − *T*_K_)^−1/^*^d^*. The different scaling of sizes from the random first order transition argument arises since the same energy function must provide the barriers and the thermodynamics. It is worth noting that Donth has obtained a similar length scale but by a different argument, which does not have a direct connection to the kinetics [36].

The larger size of these entropic droplets compared to those of Adams-Gibbs theory as the entropy crisis is approached makes them excellent candidates for explaining the dynamic heterogeneities recently discovered by Ediger through hole burning spectroscopy on molecular and polymeric glasses [37].

The simpler form of the entropic droplet argument which gives the *s*_o_^−2^ behavior has been made quantitative for the random heteropolymer globule by Takada and Wolynes [38]. Their calculations suggest that heteropolymer globules of the size of the smaller proteins would actually reconfigure as a single unit making the barriers of the order of those predicted by the GREM.

## 5. Summary and Prospects

While an old theme, entropy crises remain central to understanding the glass transition and other problems in polymer physics, such as protein folding. The model of a random first order transition seems to capture the essentials of glassy dynamics. At least for most polymers, the use of explicit randomness is justified but there is also recent progress confirming the view that randomness can be self-generated [39].

The random heteropolymer has been studied mostly for its relevance to biopolymers. This article emphasizes its relevance to atactic artificial polymers too. The finite size of protein molecules makes the mean field ideas most relevant there but the main issue for biopolymers is whether the non-random aspects of evolutionary design are actually dominant. Understanding the polymeric glass transition is an important backdrop to that discussion [40].
